# Kawasaki-like diseases and thrombotic coagulopathy in COVID-19: delayed over-activation of the STING pathway?

**DOI:** 10.1080/22221751.2020.1785336

**Published:** 2020-07-05

**Authors:** Jean-Marie Berthelot, Ludovic Drouet, Frédéric Lioté

**Affiliations:** a Rheumatology Department, Nantes University Hospital, Nantes, France; b CREATIF (centre de référence et d'éducation aux antithrombotiques d'Île-de-France); c Service de cardiologie, hôpital Lariboisière, Paris, France; d Rheumatology Department, centre Viggo Petersen, Paris, France; e Hôpital Lariboisière, Paris, France; f Université de Paris, Paris, France

**Keywords:** COVID-19, STING, Kawasaki disease, coagulopathy, thrombosis, tissue factor, ACE2, aspirin

## Abstract

We previously made the hypothesis that STING contributes to COVID-19. The present review detail new arguments for over-activation of STING pathways in COVID-19, following the description of hyper-coagulability and Kawasaki-like diseases in children. Indeed, Kawasaki disease is induced by overreaction of innate cells following exposition to various viruses, including herpes viruses which trigger STING. It predisposes to diffuse vasculitis and aneurysms, whereas STING is over-expressed in arterial aneurisms. The redness at the inoculation site of bacillus Calmette-Guérin, a specific feature of Kawasaki disease, is reproduced by activation of the STING pathway, which is inhibited upstream by aspirin, intravenous immunoglobulins, and Vitamin-D. SARS-CoV2 binding to ACE2 can lead to excessive angiotensin II signaling, which activates the STING pathway in mice. Over-activation of the STING-pathway promotes hyper-coagulability through release of interferon-β and tissue factor by monocytes-macrophages. Aspirin and dipyridamole, besides their anti-platelet activity, also reduce tissue factor procoagulant activity, and aspirin inhibits the STING pathway upstream of STING. Aspirin and dipyridamole may be used, in combination with drugs blocking downstream the activation of the STING pathway, like inhibitors of IL-6R and JAK/STAT pathways. The risk of bleeding should be low as bleeding has not been reported in severe COVID-19 patients.

## GRAPHICAL ABSTRACT

**Figure:**
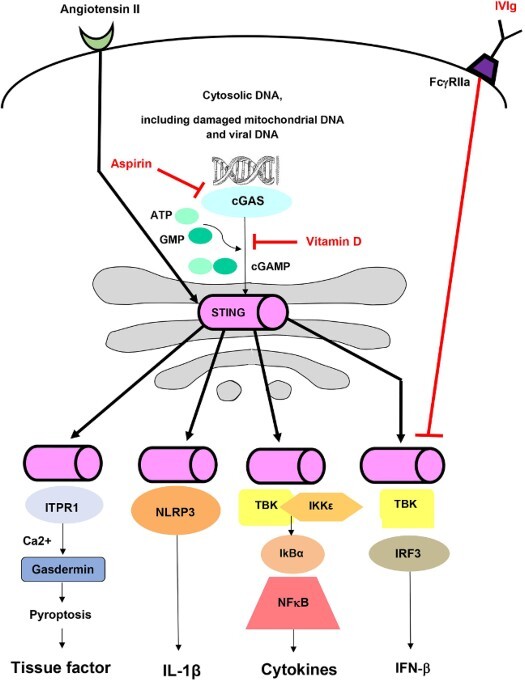

Triggering of the STING pathway by alien or self cytosolic DNA, can activate (from right to left): IRF-3 (partly inhibited by IVIgs after linking of FcγRIIa [38]), NK-κB, and the inflammasome NLRP3 [9].

This might occur even more frequently in COVID-19 patients with simultaneous enhancement of STING synthesis following excess of angiotensin II on cell membrane, secondary to the ACE1/2 imbalance (more pronounced in males), induced by the binding of SARS-CoV2 to ACE2 [42–45].

STING can contribute to Kawasaki disease, an innate overimmune response disorder following viral insults, in several ways. The mainstays of Kawasaki disease treatment, IVIgs and aspirin, both inhibit the STING pathway [38,39], which is also down-regulated by Vitamin-D [37].

Excessive triggering of the STING pathway can also lead to pyroptosis of monocyte-macrophages, and release of the highly prothrombotic tissue factor [54–57], also found in excess in lung tissues of severe COVID-19 patients [49].

## Introduction

The most severe cases of COVID-19, a disorder induced by the SARS-CoV2 coronavirus after its binding to human angiotensin-converting enzyme 2 (ACE2), combine acute respiratory distress syndrome and coagulation dysfunction, sometimes leading to visceral ischemia, and rarely to acral thrombosis [1].

Minimally invasive autopsies concluded that viral RNA was restricted to some lung cells: mainly pneumocytes II, bronchiolar epithelial cells, and macrophages [2]. However, further studies provided evidence of direct viral infection of the endothelial cells, with diffuse endothelial inflammation [3].

The pathogenesis of COVID-19 is still far from being clear, moreover as SARS coronaviruses usually do not induce severe cytotoxicity by themselves [4].

During the first days following infection, SARS-Cov viruses suppress innate immune responses to gain a window of opportunity for efficient virus replication [5]. In a second phase, in the most severe cases, the host's immune response displays a delayed over-reaction [6], including a “cytokine storm” with a high amount of IL-6 secretion. Monocytes-macrophages could be the cells most responsible for the release of those cytokines, since SARS-CoV2–infected patients often present with marked lymphopenia and harbor an expanded population of circulating monocytes that secrete both IL-6 and IL-1β [7]. However, adaptive over-response of T cells, B cells, and even epithelial and endothelial cells, could contribute to this cytokine storm.

The observation that some previously healthy children and young adults die, whereas some of the oldest COVID-19 patients have only mild symptoms, strongly supports the contribution of genetic variances to COVID-19 severity, as already extensively demonstrated for severe pneumonitis induced by other positive-strand RNA viruses [8].

Accordingly, we previously put forward the hypothesis that at risks variants of *TMEM 173*, which encodes STING, are associated with both over-secretion of IFN-β and severity of COVID-19 pneumonitis, and contribute to its pathogenesis [9].

STING, encoded by *TMEM173*, and located on endoplasmic reticulum, acts as both a direct cytosolic DNA sensor and an adaptor protein in type I interferon (IFN) and NF-κB signalling. More precisely, the cyclic GMP-AMP synthase (cGAS) protein is a main cytosolic sensor of damaged self-DNA (including mitochondrial DNA) or foreign DNA (i.e. intra-cellular bacterial and/or viral DNA, and indirectly some viral RNA). cGAS binding to DNA catalyses the production of a second messenger, cyclic GMP-AMP, which activates the STING-TBK1-IRF3 and/or NFκB signalling axis. Activation of IRF-3 leads to the synthesis of type I IFN following STAT 1/2 transcription, while activation of NF-κB leads to secretion of cytokines, including IL-6, TNF-α, and IL-1β [9] (Figure).

An argument for overactivity of the STING pathway in severe COVID-19 was that age, obesity, and diabetes, induce a marked activation of STING, and are also associated with worse prognosis of COVID-19 [9]. Another rationale was that a mutation of STING (replacement of the highly conserved and functionally important serine residue S358) is the reason why bats do not develop a delayed cytokine storm when infected by SARS-Cov viruses [9,10]. Indeed, bats are the only flying mammals, and the high metabolic demand of flight (which causes much DNA damage and the release of self-DNA into the cytoplasm), requires this high threshold for STING activation to avoid over-response. Consequently, bats do not overreact to viruses, even when damaged self-DNA, including mitochondrial DNA, accumulates in the cytoplasm, and those flying mammals have an increased capacity to co-exist with viruses, including coronaviruses, and to spread them. Reciprocally, some humans with gain of function mutations of STING, possibly different from those previously described in interferonopathies, might over-react to damages induced in cells by SARS-CoV2 infections [9]. The precise mechanisms of this delayed activation of STING by RNA virus need to be further studied [10,11], but at least two explanations have been given: first, fusion between viral envelopes and target cells specifically stimulates a type I interferon response which is dependent on STING, but independent of DNA, RNA and viral capsid [12]; this mechanism appears improbable in COVID-19, at least during the first steps of infections, since, on the opposite, SARS-CoV viruses PLpro proteases inhibit association with the signalling complexes assembled around STING, and block downstream signalling via IRF-3 [13]; 2-SARS-CoV induce a delayed raise of cytosolic DNA due to intracellular damages [11,14], which would better explain the delayed onset of the cytokine storm of COVID-19, by a rebound effect of innate immunity (especially if the initial inhibition of STING by PLpro does not last more than several days). This delayed increase in cytokine secretion by innate cells might combine with the simultaneous raise of the adaptive T and B cell immune response, a possible second source of deferred cytokine release.

The hypothesis of a central role of STING activation in COVID-19 has been reinforced by: 1-the observation that STING is mostly expressed by endothelial cells and pneumocytes type II [15], i.e. exactly the two main targets of SARS-CoV2; 2-recent reports claiming that drugs blocking IL-6 [16] and STAT-1 [17] downstream of the STING pathway, gave promising results in open trials performed in intensive care units (ICUs); 3-the recent finding that type I IFNs, and to a lesser extent type II IFNs, upregulate ACE2 in human airway epithelial cells, including type II pneumocytes [18]. As binding of SARS-CoVs leads to ACE2-receptor-mediated internalization, an over-production of IFNs due to gain-of-function variants of STING should promote the ability for SARS-CoV2 to maintain cellular targets in human upper airway epithelial cells [18]. This forward feed-back loop might also account for the paradoxical increased severity of SARS-CoV infections following administration of IFNβ, as also observed in mice models [19].

The present review aims to focus on two other possible consequences than pneumonitis of over-activation of the STING pathway in severe COVID-19, at least in patients with at risk TMEM173 variants, namely: 1-the severe and sometimes lethal Kawasaki diseases (KD) described in some young patients with COVID-19 [20], since several arguments suggest that KD might also be fostered by over-activation of the STING pathway; 2-the frequent resistance of the poor prognosis thrombotic coagulopathy of COVID-19 to thrombo-prophylaxis by heparin or factor Xa inhibitors. This resistance could partly result from the excessive release of interferon-β and tissue factor (TF) following the delayed over-activation of the STING pathway in the second phase of SARS-CoV2 infection. Indeed, excessive release of both interferon-β and TF can also induce a severe hyper-coagulability. Those observations might prompt trials to study the usefulness of adding aspirin to anti-Xa or heparin in severe COVID-19, as also successfully performed previously in patients with past myocardial infarction.

### Kawasaki disease (KD) and COVID-19 both predispose to diffuse vasculitis with thrombosis

Kawasaki disease (KD) is a systemic vasculitis of unknown aetiology which predominantly affects medium and small-sized muscular arteries, mostly in children, and much less frequently in adults. KD is associated with the delayed occurrence of arterial aneurysms: coronary aneurysms (up to giant coronary aneurysms [21]) in roughly 30% of untreated patients, and systemic arterial aneurysms in 2%. Asymptomatic cerebral vasculitis is also more common in KD than previously believed, and cerebral aneurysms have been reported, although much less frequently than coronary aneurysms.

Vasculitis has similarly been described in COVID-19, including chilblains-like lesions. Histology from one case showed signs of vasculitis with evident fibrin thrombus [22]. Of note, familial chilblains lesions have been ascribed to a heterozygous gain-of-function mutation in STING [23]. This is the first argument for a contribution of over-activation of the STING pathway to COVID-19 with acral necrosis. A stronger argument is the observation that COVID-19 features (interstitial pneumonitis, with inflammatory vasculopathy up to acral necrosis, and marked lymphopenia) are very similar to those of SAVI syndromes (STING associated vasculopathy with onset in infancy) induced by gain of function mutations of *TMEM173* [24].

The recent description of KD-like in young patients with COVID-19 [20] may prompt follow-up of all children with COVID-19 to seek for delayed expression of coronary and/or cerebral endothelial lesions, including small aneurysms, as sequelae of vasculitis induced by SARS-CoV2. Similar follow-up could be useful in adults, moreover as an increased rate of strokes has already been reported in adults with COVID-19 [25], which might not only result from hypercoagulability, but could also indicate similar underlying cerebral vasculitis.

### Kawasaki disease has been attributed to genetically encoded over response of the innate immune system to viral infections

Global investigation of immune repertoire in KD strongly suggests infectious causes rather than adaptive autoimmune, since the B-cell selection phenomenon has a non-autoimmune pattern. KD probably results from the exposure of a genetically predisposed individual with aberrant innate immune system to various pathogens-derived PAMPs or DAMPs [26].

Those pathogens are presumed to be mainly viruses, since the immune transcriptional profile in KD coronary artery tissues has features of an antiviral immune response (such as activated cytotoxic T lymphocyte and type I IFN-induced gene up-regulation) [26]. Increased plasma level of CXCL10, a representative IFN-α2α/γ-inducible protein is a promising biomarker for the early acute phase of KD [27], and is also a prognosis factor in COVID-19.

Numerous viruses have been described as possible triggers of KD, including coronaviruses (strains 229E, HKU1, NL63, OC43), although rarely identified (< 5%) [20]. Herpes virus, including HHV-6 and -7 have been more frequently reported as triggers of KD [28], and, interestingly, they also activate the STING pathway. Upon HSV infection and cytosolic DNA stimulation, STING binds to NLRP3 and promotes the inflammasome activation and IL-1β release, a cytokine critically required in mouse models of KD [29].

Whereas redness at the inoculation site of bacillus Calmette-Guérin (BCG) has been considered as a feature of KD, herpes virus, including HHV-6, can also cause skin lesions at the BCG inoculation site [30]. This phenomenon could be explained by the re-activation of the STING pathway in innate and/or stem cells previously trained by BCG to adopt epigenetic changes following a first exposure to mycobacterial DNA. Indeed, a recombinant BCG released high levels of a STING agonist, and lead to an enhanced synthesis of IFN-β, IL-6 and IL-1β [30,31].

Damaged self-DNA, due to viral infections or other triggers, is another major activator of the STING pathway. The report of a 17-month-old Japanese female who was hospitalized for KD following severe sunburn all over her body, with high levels of the DAMP HMGB1, and infection ruled out as a cause, also supports the hypothesis that STING activation contributes to KD [32] in young patients with at risk *TMEM173* variants.

### Prevention of STING over-activation could lower the risk of delayed arterial aneurysms

Over-activation of the STING-pathway, could increase the risk of delayed aneurysms not only in KD but also COVID-19 vasculitis. Indeed: 1-even incomplete form of KD (KD-like disorders) can lead to severe, and sometimes lethal, delayed aneurysms; 2- the STING pathway is activated in aortic aneurysms, especially those induced by infections, and it contributes to their pathogenesis [33]. As giant coronary aneurysms in children with KD can regress following the use of anti-cytokines [21], drugs inhibiting the cytokine storm occurring in severe COVID-19 might be also useful to prevent the delayed onset of similar coronary or cerebral aneurysms, either in children with KD-like features and/or adults with COVID-19.

### Vitamin-D, which mitigates both KD and COVID-19 severity, also represses the cGAS/STING/IFN cascade

The 25(OH)-vitamin D is crucial in the regulation of immunologic processes. In a study of 79 children with KD, levels of 25(OH)-vitamin D were much lower than in controls (9.17 ± 4.94 vs 23.3 ± 10.6 ng/mL, *p* < 0.0001), especially in the subgroup who developed coronary artery abnormalities (4.92 ± 1.36 vs 9.41 ± 4.95 ng/mL, *p* < 0.0001). Serum 25(OH)-vitamin D levels correlated with inflammation and both coronary artery aneurysms (*p* = 0.005) and non-aneurysmatic cardiovascular lesions (*p* < 0.05) [34]. Low 25(OH)-vitamin D is also associated with poor COVID-19 outcome [35]. This correlation might contribute to the lower than expected population mortality from COVID-19 in countries south of latitude 35 degrees North [36]. Although an increase rate of cathelcidin has been postulated to be one of the mechanisms through which vitamin-D could better control SARS-CoV2 initial infection, vitamin-D might also prevent the delayed over-reaction of the immune system (Figure). Indeed, in prematurely senescent cells, calcitriol (1,25-(OH)_2_ D_3_) represses the cGAS/STING/IFN cascade induced by exposure of chromatin self-DNA in the cytosol [37].

### Aspirin and IVIgs inhibit STING

The reasons why aspirin and intra-venous immunoglobulins (IVIgs) are the mainstays of KD treatment are still obscure [20]. Interestingly, it was recently shown that both treatments inhibit (upstream) the STING pathway, whereas aspirin also lowers (downstream) TF pro-coagulant activity.

IVIgs and aspirin are the two mainstays of KD treatment, and both dampen the STING activation pathway upstream of STING (Figure).

In human myeloid immune cells, IgG induced a selective inhibition of TLR, RIG-I-like receptor, and of STING-dependent type I and III IFN gene transcription. This type I and III IFN suppression was mediated by Syk and PI3 K independent inhibitory signalling via FcγRIIa [38].

Whereas the mechanisms through which aspirin is effective to reduce clinical activity of KD and prevent coronary aneurysms are still considered as unclear, inhibition of the STING pathway activation by aspirin could contribute to explain both. Indeed, activation of the STING pathway has been observed in human aneurysms, and aspirin directly acetylate cGAS on either Lys384, Lys394, or Lys414. This contributes to keeping cGAS inactive, and robustly suppresses self-DNA-induced autoimmunity through the STING pathway [39].

### Sequencing of genes of the STING pathway could be performed in Kawasaki diseases to seek for associated variants shared with those found in severe COVID-19

Pointers that suggest a genetic basis of KD include a high disease prevalence in North-East Asian populations, a high risk among siblings, and familial occurrence of cases. Although *TMEM173* has not been so far listed among the candidate genes to account for familial clustering of KD, sequencing of *TMEM173* and other genes contributing to the STING pathway may be informative in KD and conditions with KD-like features, including COVID-19. Some patients with COVID-19, but also KD, might indeed over-react to various cytosolic DNA damage and/or viral triggering, due to *TMEM173* variants (and/or variants of genes encoding for other molecules of the STING pathway), associated with worse prognoses.

### Kawasaki disease also increases the risk of thrombosis

Analyse of coagulation function in 42 children with KD prior to IVIg treatment indicated an overall hyper-coagulability with well-balanced dynamic coagulation and fibrinolysis [40]. Non-responders to IVIg remained hyper-coagulable after primary treatment [40], but the coronary aneurysms resulting from vascular inflammation is an even greater source of further thrombosis. Indeed, although they often may remain clinically silent until adulthood, even small to moderate-sized aneurysms that seem to normalize on echocardiography in childhood can lead to stenosis and thrombosis decades after the acute illness [41].

### The binding of SARS-CoV2 to ACE2 could directly activate the STING pathway, at least in cardiovascular cells

Physiologically, ACE2 degrades angiotensin II, the master regulator of the renin-angiotensin-aldosterone system, thereby converting it into vasodilatory molecules. SARS-CoV2, following proteolytic cleavage of its S protein by a serine protease, binds to the transmembrane ACE2, a homologue of ACE, to enter type 2 pneumocytes, macrophages, perivascular pericytes, and cardiomyocytes [42]. In heart failures, an enhanced shedding of ACE2 might account for the increased plasmatic concentrations of ACE2 observed in men as compared to women [43].

In COVID-19, an ACE1/ACE2 imbalance occurs, due to the binding of SARS-CoV2 to ACE2, reducing ACE2-mediated conversion of angiotensin II to angiotensin peptides that physiologically counteract pathophysiological effects of ACE1-generated angiotensin II [42]. This imbalance could be even more pronounced in aged patients or those with either obesity, diabetes, or cardiac insufficiency, since all those conditions are already associated with a basal lower expression of ACE2 on cells [44].

Interestingly, it has been shown on cultured murine myocardial cells that addition of angiotensin II induced a strong expression of STING, this expression being still accelerated by endoplasmic reticulum stress activator [45]. Therefore, excessive angiotensin II signaling in COVID-19 due to poor ACE2 conversion of angiotensin II at the cell surface, could contribute to activate the STING pathway (Figure). This activation might be still enhanced by the release of oxidized mitochondrial DNA following polymorphonuclear NETosis induced by viral or intracellular bacterial (co)infection. Indeed, when injected into mice, this oxidized mitochondrial DNA also activates the STING pathway [46].

### Thrombotic coagulopathy is associated with poor COVID-19 outcome

Thrombotic coagulopathy contributes to the severity of COVID-19 infection, and excess of venous, arterial, or microvascular associated events. Venous thrombo-embolism was observed in 27%, and arterial thrombotic events in 3.7% of COVID-19 patients admitted in ICU, and severe coagulation abnormalities occur in almost all critical COVID-19 cases [47]. Coagulation dysfunction is common in patients with COVID-19, especially fibrinogen and D-dimer elevation. Studies using thrombo-elastography or thrombo-elastometry supported hyper-coagulability induced by a severe inflammatory state, whereas D-Dimers reflect the activation of coagulation, which can even lead to acute disseminated intravascular coagulation [48].

In the lung, hyaline thrombi have been found during autopsies in blood vessels of alveolar septum. A more consistent finding in acute respiratory distress syndrome of COVID-19 is the deposition of fibrin in the lung parenchyma [49]. Tissue factor (TF), the high-affinity receptor for coagulation factor VIIa, is exposed on damaged alveolar endothelial cells and on the surface of leukocytes, promoting activation of the coagulation pathway, leading to fibrin deposition [49].

Arterial thrombosis with a high thrombosis load is also a common feature of COVID-19 infection. Myocardial ischemia with an elevated troponin level occurred in 7–17% of patients hospitalized with COVID-19, and 22–31% of those admitted to the ICUs. An increase rate of ischemic stroke [25] and limb acute ischemia has also been reported, up to distal necrosis, as also observed in some genetic disorders with over-activation of the STING pathway [50]. Those COVID-19 patients present with severe acro-ischemia, manifesting as finger/toe cyanosis, skin bulla and dry gangrene. Although low molecular weight heparin treatment decreased D-dimer and fibrinogen degradation products, significant improvement in clinical symptoms was not observed [51].

Therefore, understanding the precise mechanisms of hypercoagulability in COVID-19 is urgently needed [48].

Although lupus anticoagulant (found in roughly 20% of severe COVID-19) [52], and complement-mediated thrombotic microangiopathy might fuel some of those thrombosis, several evidence suggest that overactivation of the STING pathway also contributes to the hypercoagulability observed in COVID-19.

### Overactivation of the STING pathway promotes hypercoagulability through over-expression and release of IFN-β and tissue factor

Among the first observations supporting the hypothesis that polymorphisms in the STING pathway contribute to the pathogenesis of severe COVID-19 [9], was listed the demonstration that the mitochondrial damage-cGAS-STING-IRF3 pathway is critically involved in metabolic stress-induced endothelial inflammation [53]. Such endothelitis might be a first explanation for the difficult to treat thrombosis observed in severe COVID-19.

However, there is also evidence that activation of the STING pathway more directly over-activates coagulation.

First, STING activation markedly increases the release of IFN-β, whereas excess of IFN-β can induce thrombotic microangiopathy. In bacterial sepsis, type 1 IFN also mediates infection-induced disseminated intravascular coagulation, by amplifying the release of high-mobility group box 1 (HMGB1) into the bloodstream, which markedly increases the pro-coagulant activity of TF [54].

Second, in severe COVID-19, IFN-β might not be the sole explanation for the hyper-coagulation and pro-coagulant activity of TF. Indeed, a recent work showed that activation of STING in monocytes-macrophages during sepsis can also induce hyper-coagulability through the release of TF independently of IFN secretion [55]. The pyroptosis pathway used (STING-ITPR1-calcium release-gasdermin D-pyroptosis-TF) does not rely on the classical components of the STING pathway. Whether similar release could also occur in pyroptotic endothelial cells has not been addressed, but appears plausible since: 1-pyroptosis has also been described in endothelial cells; 2-patients with COVID-19 have elevated levels of lactate dehydrogenase, a marker of pyroptosis [56]; 3-inflammasome activation, which is also triggered by STING, can lead to pyroptosis and TF release following other innate over-immune responses associated with tissue damages [57].

The observation that STING can activate coagulation independently of the cGAS-STING-IRF3 pathway might prompt to combine other drugs with anti-cytokines to prevent thrombosis in the worse cases. The release of TF in lung and vessels following STING over-activation might indeed be a critical event in severe COVID-19, and an important target for other treatments than anti-cytokines, moreover as genetical or pharmacological inhibition of TF abolishes inflammasome-mediated blood clotting, and protects against death [57].

### Usual thrombosis prophylaxis is often not sufficient to prevent thrombotic coagulopathy in severe COVID-19

In the retrospective study of 184 Dutch patients with proven COVID-19 pneumonia admitted to an ICU, only 17 (9.2%) had therapeutic anticoagulation at admission, whereas coagulopathy was already present in 70 (38%) [47]. Once in ICU, all patients received at least standard doses thrombo-prophylaxis by enoxaparin and/or nadroparin, but none has been given antiplatelet drugs. Subsequently, and despite this systematic thrombo-prophylaxis, the 31% incidence of thrombotic complications remained remarkably high, although none of those patients developed diffuse intra-vascular coagulation [47]. Therefore, addition of treatment dampening the TF pro-coagulant activity could be helpful.

### Aspirin and dipyridamole lower tissue factor pro-coagulant activity and might be added to thrombo-prophylaxis in severe COVID-19

Previous studies showed that TF pro-coagulant activity declined following treatment with clopidogrel alone, and with combinations of clopidogrel with aspirin, whereas no changes were noted in plasma factor VIIa [58].

Dipyridamole, through its adenosine effect, is an inhibitor of platelet aggregation, but it also blocks the lipopolysaccharide induced increase in monocyte-associated TF activity. It has been proposed to treat COVID-19 by dipyridamole on the basis of *in vitro* suppression of SARS-CoV2 replication, and a proof-of-concept trial involving 31 patients with COVID-19 [59]. However, dipyridamole, a purinergic modulator, also increases cyclic adenosine monophosphate (cAMP) and cyclic guanosine monophosphate (cGMP) [60], which might simultaneously fuel STING activation (Figure), minimizing its usefulness.

Aspirin, which inhibits the activation of the STING pathway upstream, through acetylation of cGAS, might also be tested to treat severe COVID-19, since it should both inhibit the TF-induced hyper-coagulation associated with COVID-19, and contribute to slow down the cytokine storm that can be induced by over-activation of the STING pathway. Indeed, aspirin keeps cGAS inactive, and robustly suppresses self-DNA-induced autoimmunity through the STING pathway [39].

The mechanisms of COVID-19 hypercoagulability might significantly differ from conditions in which heparin and factor Xa inhibitors perform better (and/or induce less bleeding than aspirin). Therefore, the usefulness of dipyridamole and low-dose aspirin in preventing the deadly consequences of hypercoagulability in lung and/or heart and/or peripheral arteries of severe COVID-19 patients, is an important point to address. During their hospital stay, low-dose aspirin could be added to heparin and/or factor Xa inhibitors, since: 1-in TF-triggered blood under conditions of flow, platelet inhibitors maintain their aggregation-inhibiting effect at sites of thrombin formation [61]; 2-bleeding manifestations, even in those with disseminated intravascular coagulopathy have not been reported so far in severe COVID-19 patients [62]; 3- In the COMPASS trial on patients with stable coronary artery disease, 100 mg daily aspirin alone did not performed better than 5 mg daily rivaroxaban (a factor Xa inhibitor), alone, but the addition of aspirin to rivaroxaban was significantly more efficient to prevent the primary outcome (myocardial infarction, stroke, or cardiovascular death). More bleeds were seen in the rivaroxaban alone group than in the aspirin alone group (3% versus 2%) (*p*<0·0001) whereas combination of rivaroxaban and aspirin did not increased the rate of bleeding (3%), and deaths were overall reduced by 23% when aspirin was added to rivaroxaban [63]. However, such addition of aspirin or dipyridamole might be efficient only in patients also treated by drugs blocking the STING pathway downstream, like inhibitors of IL-6R [16] and JAK/STAT [17] pathways.

## Conclusion

Numerous arguments suggest that a delayed over-activation of the STING pathway contributes to the pathogenesis of COVID-19. Severe COVID-19 might result from a rebound effect of the innate immune response following accumulation of self-DNA damage within the cytosol and triggering of STING, combined with the simultaneous raise of the adaptive immune response. Other observations also support a role of STING to some features of vasculitis, like those associated with KD, and STING might also contribute to the delayed onset of arterial aneurysms observed in severe KD. Association of variants of *TMEM173,* encoding STING, and of other genes regulating the STING pathway, with COVID-19 severity, might be searched, especially in patients with vasculitis and/or KD-like features. Drugs down-regulating the STING pathway either upstream (like aspirin and IVIgs, and Vitamin -D), or downstream (like IL-6 inhibitors or inhibitors of the JAK-STAT pathway) could be beneficial in COVID-19, and perhaps also in some KD. Since STING also activate coagulation through another independent pathway (STING-ITPR1-calcium release-gasdermin D-pyroptosis) which can lead to TF deposition in various tissues, low-dose aspirin or dipyridamole could be added to other STING inhibitors to better prevent the thrombotic coagulopathy of COVID-19 patients in patients with biological signs of coagulation dysfunction. Better examining the contribution of the STING pathway to the thrombotic coagulopathy associated with COVID-19 could help and better treat deadly vasculitides induced by other viruses.
